# *In situ* tomographic study of a 3D-woven SiC/SiC composite part subjected to severe thermo-mechanical loads

**DOI:** 10.1107/S1600577522000406

**Published:** 2022-01-27

**Authors:** Léonard Turpin, Stéphane Roux, Olivier Caty, Andrew King, Sébastien Denneulin, Éric Martin

**Affiliations:** a CNRS/CEA/SAFRAN/Univ. de Bordeaux, LCTS – Laboratoire des Composites Thermo-Structuraux, F-33600 Pessac, France; b Univ. Paris-Saclay/ENS Paris-Saclay/CNRS, LMT – Laboratoire de Mécanique et Technologie, F-91190 Gif-sur-Yvette, France; c Safran Ceramics, F-33700 Mérignac, France; d Synchrotron SOLEIL, F-91192 St Aubin, France

**Keywords:** X-ray micro-tomography, image processing, ceramic matrix composite (CMC), thermo-mechanical loading, multi-axial loading

## Abstract

A high-temperature multi-axial test is carried out to characterize the thermo-mechanical behaviour of a 3D-woven SiC/SiC composite aeronautical part under loads representative of operating conditions. The sample is L-shaped and cut out from the part. It is subjected to severe thermal gradients and a superimposed mechanical load that progressively increases up to the first damage. The sample shape and its associated microstructure, the heterogeneity of the stress field and the limited accessibility to regions susceptible to damage require non-contact imaging modalities. An *in situ* experiment, conducted with a dedicated testing machine at the SOLEIL synchrotron facility, provides the sample microstructure from computed micro-tomographic imaging and thermal loads from infrared thermography. Experimental constraints lead to non-ideal acquisition conditions for both measurement modalities. This article details the procedure of correcting artefacts to use the volumes for quantitative exploitation (*i.e.* full-field measurement, model validation and identification). After proper processing, despite its complexity, the *in situ* experiment provides high-quality data about a part under realistic operating conditions. The influence of the mesostructure on fracture phenomena can be inferred from the tomography in the damaged state. Experiments show that the localization of damage initiation is driven by the geometry, while the woven structure moderates the crack propagation. This study widens the scope of *in situ* thermo-mechanical experiments to more complex loading states, closer to in-service conditions.

## Abstract 2

An *in situ* corner bending test under a high thermal gradient is followed by both computed tomography and thermography. Experimental procedure, image processing and preliminary conclusion are presented.

## 1. Introduction

3D-woven ceramic matrix composites (CMCs) made with silicon carbide matrices reinforced by silicon carbide fibres are expected to replace super-alloys in the hot parts of the next generation of aircraft engines, namely for GE/SAFRAN LEAP. Their outstanding structural properties at very high temperatures make them promising materials (Murthy *et al.*, 2008 ▸). Due to its complex microstructure, a 3D-woven composite part is more resistant to delamination and impacts (Boussu *et al.*, 2015 ▸). Its architecture is designed to comply with the overall part geometry and loading. The SiC/SiC CMCs are designed to approach the thermo-mechanical behaviour of a pure ceramic in the elastic domain while displaying a progressive failure. A precise and reliable characterization of its crack mechanics under complex multiaxial thermo-mechanical loading is crucial to ensure the safety of aeronautical parts (Zok, 2016 ▸).

Damage mechanisms of SiC/SiC have been studied experimentally. At room temperature, loaded in uniaxial tension, matrix cracks occur in transverse tows or matrix-rich areas (Morscher, 2004 ▸). Within tows, cracks initiate as a coalescence of micro-debonding at the interface between fibre and matrix (Sevener *et al.*, 2017 ▸). Cracks then propagate in tows until they eventually percolate into larger through-thickness matrix cracks (Maillet *et al.*, 2019 ▸). When oxidation phenomena are negligible, damage behaviour at high temperatures is qualitatively similar (Mazars *et al.*, 2017 ▸). Bernachy-Barbé *et al.* (2015 ▸) tested tubes under combined internal pressure and traction. Since the composite is subjected to multiaxial loading, cracks mainly propagate perpendicularly to the maximum principal stress. If the load is applied off-axis from the main fibre direction, there are no transversely oriented tows, and the matrix-cracking stress is higher than for on-axis loading (Morscher *et al.*, 2007 ▸). Cracks initiate in the matrix-rich area, in the vicinity of free surfaces, and then propagate within the tows where they are deviated, generating fibre debonding and fibre ruptures (Mazars, 2018 ▸). However, current studies are mostly limited to uniaxial configurations or homogeneous loads. Complex-shaped specimens submitted to multiaxial and heterogeneous stress states have not been studied at the microscopic scale.

One of the primary purposes of current research on CMCs is to assess their ultimate loads under (or close to) operating conditions. Industrial parts have a complex mesostructure: the 3D weaving of the SiC tows has to be tailored to bent parts, curved sections or junctions. In the engine, such parts are subjected to a very intense thermal flux from combustion, and strong cooling to limit high temperatures. This competition over small distances gives rise to a very heterogeneous temperature field with very high gradients. More than the elevated temperature per se, the latter leads to large strain incompatibilities responsible for a large part of the resulting stresses. Local stresses are thus the result of (large-scale) structural thermo-mechanical loading, mesoscale details of the weaving, and potentially of micro-scale defects such as tiny pores. It is, therefore, crucial to carry out experiments that deviate from the standard practice of thermo-mechanical test design (*e.g.* seeking for the most homogeneous conditions as possible). In addition, critical zones where damage is likely to occur may be confined to relatively small regions, which may well be difficult to access. An experimental study of failure loads is essential to secure part design and is highly challenging from the instrumentation viewpoint. The present paper reports on the successful execution of such an experiment referred to as a *corner bending test*.

Compared with other non-destructive measurement devices that only give access to surface information [*e.g. in situ* optical or electronic microscopy tests (Jordan *et al.*, 2021 ▸)] or partial volume information [*e.g.* acoustic emission (Maillet *et al.*, 2019 ▸)], X-ray computed micro-tomography (CT) provides highly spatially resolved volumetric information. Hence, CT is a perfect candidate to detect the complex local morphology of the cracks. CT consists of computing a volume (3D) image from a collection of 2D radiographs acquired under different angles (Kak & Slaney, 2001 ▸; Kalender, 2006 ▸). As such, it may provide a very detailed visualization of the woven microstructure and possibly reveal the presence of porosities or micro-cracks. When performed on *in situ thermo-mechanical tests*, this imaging technique may be combined with digital volume correlation (DVC) to yield a further invaluable piece of information, namely the 3D displacement field induced by the loading (Buljac *et al.*, 2018 ▸).

*In situ* experiments in a tomograph generally require dedicated testing devices (Bale *et al.*, 2013 ▸; Maire *et al.*, 2020 ▸). Indeed, the sample has to be accessible within the most extensive possible angular range for CT (ideally 180/360°). The size and the weight of the device have to be limited to be compatible with the tomograph. In the present experiment, the applied thermal loading is very inhomogeneous and must be measured concurrently. If the coupling of two full-field measurement modalities (temperature and kinematic fields) has already been performed in 2D (Archer *et al.*, 2020 ▸), to the best of the authors’ knowledge, this is new for 3D fields. The loading device must fulfil demanding requirements for both tomographic and infrared (IR-)thermographic imaging. These requirements lead us to design an *in situ* loading device: a rotating loading gantry. Two steel columns make a rigid frame around the sample to support mechanical loads. They partially mask some projection directions during the tomographic acquisitions. Hence, tomographic reconstruction requires a bespoke algorithm (Turpin *et al.*, 2020 ▸) and specific attention to the artefacts resulting from missing information.

The present paper aims to show that finely resolved tomographic images can be obtained during an experiment wherein a complex-shaped sample is subjected to inhomogeneous thermal and mechanical loads despite the difficulties mentioned above. The methodology involves a combination of techniques detailed in the following, emphasizing the challenging points. Applications of such a procedure are broad. The authors propose some guidelines to couple measurement modalities and to process data. A corner bending test is presented as an example and proof of feasibility. Quantitative exploitation of the images to validate the mechanical model of the material will be the focus of a companion paper (Turpin *et al.*, 2022 ▸). In Section 2, the dedicated testing machine is introduced together with its limitations for tomographic acquisitions. Section 3 is devoted to the processing of radiographs to achieve good quality tomographic reconstructions. In Section 4, tomograms computed at different stages of the test are shown to provide very rich and resolved information, giving new insight into the capacity of these complex parts to sustain the extreme loading of their service conditions. After summarizing the main steps of the experiment, the concluding section[Sec sec5] gives some perspectives on the quantitative exploitation of these data for mechanical modelling.

## 2. Experimental procedure

This study focuses on corner bending tests which aim to study a structural element under coupled shear and flexion loading in a region where the woven preform has been strongly strained to accommodate its final shape (Bénézech & Couégnat, 2019 ▸). Two *in situ* tests were carried out: one at room temperature and one under a severe thermal gradient.

**Figure 1:**
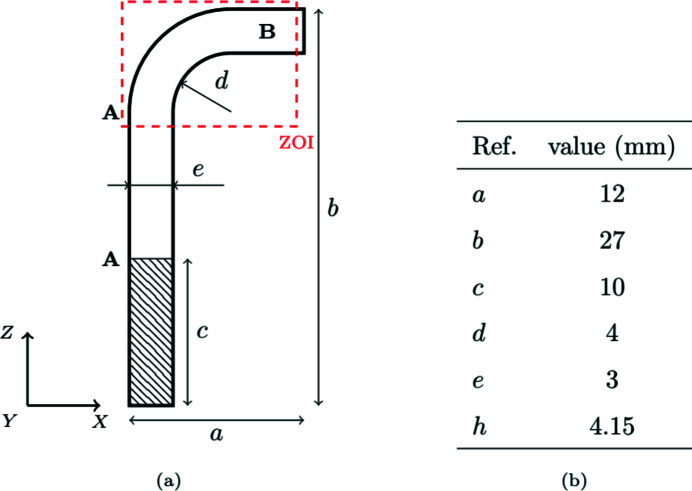
Size of the corner sample. (*a*) The hatched area is the clamping zone. The zone of interest (ZOI) corresponds to the tomographic field of view. The outer surface of the long arm **A–A** is hot, whereas the short arm **B** is cold. (*b*) *h* is the sample width in the direction perpendicular to the view plane in (*a*).

Fig. 1 ▸ details the overall sample geometry. This ‘L-shape’ is a compromise considering tomographic constraints: the sample is large enough to observe a relevant part of its mesostructure but small enough to perform acquisitions with a good resolution. It is made out of Cerasep A600^®^. Developed by Safran, Cerasep A600^®^ is one of the latest SiC/SiC composites (Halbig *et al.*, 2013 ▸; Katoh *et al.*, 2014 ▸; Kopeliovich, 2014 ▸; Wang *et al.*, 2019 ▸). This sample is built from a planar 3D-woven fabric shaped into a mould. Therefore the tows are in tension or compression, respectively, in the bent part outer or inner free surface. During forming, tows also become misaligned in the arms of the piece. The fibre density is thus not homogeneous, and the part displays a strong, spatially varying, anisotropy. The matrix is then synthesized to fill in the porosity between the tows. The two major processing steps are chemical vapour infiltration (Naslain *et al.*, 1989 ▸) and melt infiltration (Corman & Luthra, 2005 ▸).

**Figure 2:**
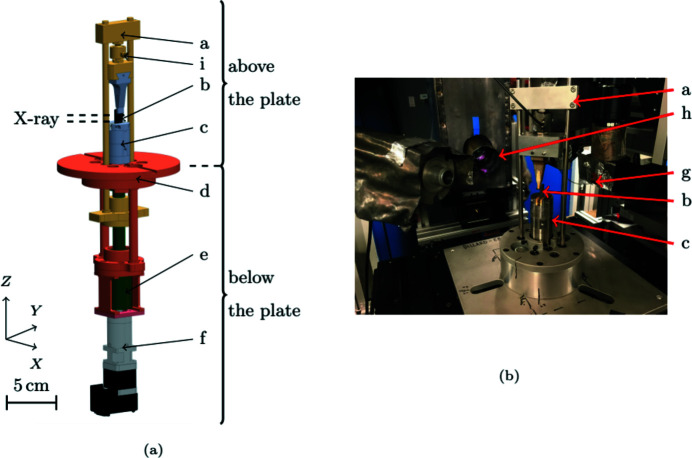
Experimental device: (*a*) CAD-rendering. (*b*) Implementation at the PSICHE beamline (King *et al.*, 2016 ▸). The sample *b* is clamped in the lower jaw *c*. Load is applied by a gantry *a* which passes through the frame *d* clamped to a tomograph rotating plate so that the actuator *e* + *f* is deported under the plate to reduce unbalance and free the observation zone. *g* is the tomograph scintillator, *h* is the infrared camera and *i* the force sensor.

**Figure 3:**
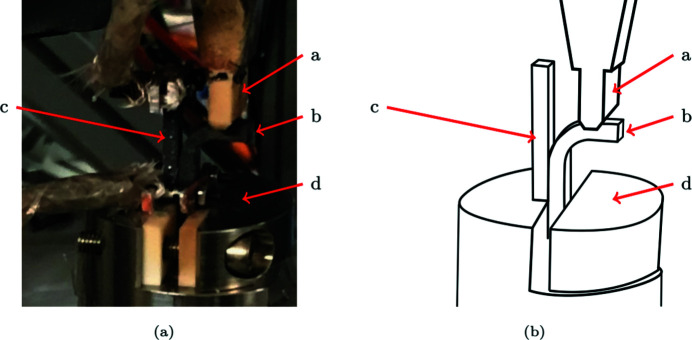
Thermo-mechanical loading of the sample. The punch *a* loads the sample *b* in flexure. The SiC/SiC resistor *c* induces the thermal gradient. *d* is the clamping jaw.

Fig. 2 ▸ shows the *in situ* testing device. The frame (d) is anchored onto the tomograph rotation stage interface plate. The sample (b) is clamped to the lower clamping jaw (c). The loading gantry (a) moves up and down thanks to an actuator located under the interface plate, consisting of the worm screw (e) and geared motor (f). The long arm of the sample is clamped in the region hatched in Fig. 1 ▸, and an alumina punch loads the short arm (Fig. 3 ▸).

The load is measured thanks to a force sensor [HBM-U9C, (i) in Fig. 2 ▸]. The maximum load is 20 kN (in traction or compression). Due to the shape of the sample, the force sensor is not submitted to pure traction/compression but also flexure. It was designed considering this flexure loading. The force signal is filtered using an analogue 100 Hz Bessel filter to reduce the noise induced by the magnetic environment of the tomograph. The force uncertainty is 4 N.

A SiC/SiC resistor is fixed on the hot face of the sample (Figs. 1 ▸ and 3 ▸). During the test, it is maintained at about 1000°C. Coupled with radiative and convective cooling of other free surfaces and the contact with the punch, it induces a substantial thermal gradient within the sample. The thermal loading is applied before the first mechanical loading step.

The test is controlled in displacement. Stepwise loading is necessary to perform tomographic acquisitions, which require about 15 min each. When the tensile force reaches prescribed values, the displacement is held constant and a tomographic scan is acquired. Between two steps, the loading is applied monotonically and quasi-statically (0.2 mm min^−1^). Fig. 2 ▸(*b*) illustrates the implementation of the loading device at the PSICHE beamline. The X-ray beam axis [normal to the detector (g)] and the IR camera (h) optical axis are perpendicular to each other.

The tests were carried out at the PSICHE beamline at Synchrotron SOLEIL (King *et al.*, 2016 ▸). A pink beam centred around 40 keV was used. The working distance was about 120 mm, a 1.05 mm thick copper filter and an X-ray mirror (as a low-pass filter) were used to define the beam spectrum. Acquisitions were performed with a voxel size of 3.14 µm over 360° with an offset rotation axis. The reconstructed field of view was 12.40 mm × 12.40 mm × 3 mm. Five thousand nine hundred projections were acquired per scan with an exposure time of about 50 ms each. The scan duration was thus about 5 min. Three overlapping scans were stitched together along the vertical direction to increase the field of view to 7.1 mm.

**Figure 4:**
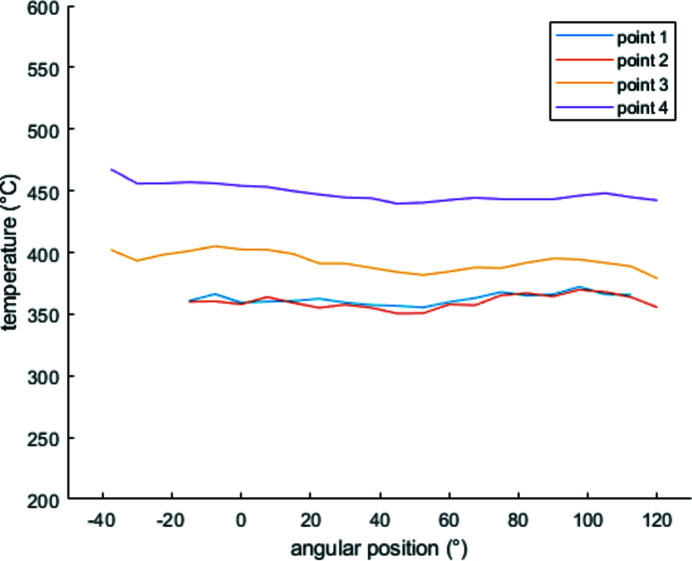
Evolution of the temperature during the rotation at several points on the sample. Each point is hidden during one half of the rotation and by the columns. Values at grazing angles are not considered. Positions of the considered points are plotted in Fig. 5 ▸

The thermal field was measured thanks to an IR camera (FLIR SC7210-7500). Thermograms and tomographic scans were acquired simultaneously. The rotation of the sample provides the thermal field under the whole angular range, assuming the temperature field is stable in time. The camera memory was sufficient to acquire one and a half turns. The stability assumption was first experimentally tested, computing the difference between the same angular position images of each full rotation and checking that it remained negligible. Then, following the temperature of several material points confirmed the stability during the rotation (Fig. 4 ▸). A bi-chromatic pyrometer measured the temperature of a hot spot on the resistor surface to check and monitor the value of the temperature. This temperature was also used to calibrate the temperature field acquired by the IR camera.

**Figure 5:**
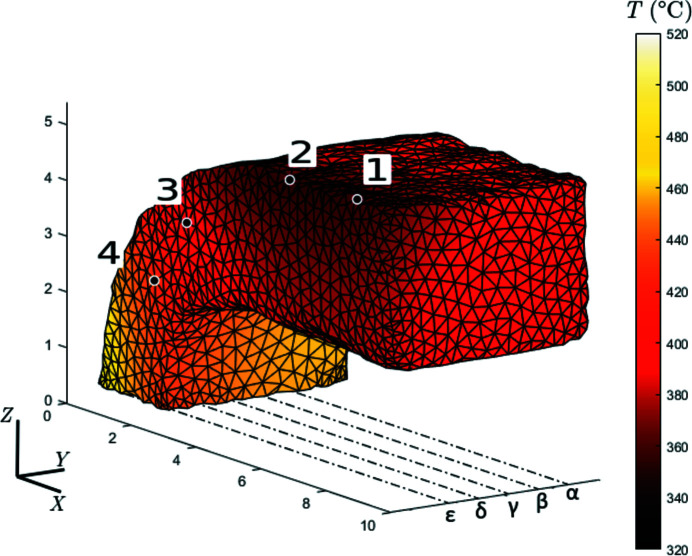
Reprojection of the thermal field measured at the initial step. The thermal gradient along the *X* direction is representative of operating conditions. The points in black surrounded by white correspond to the points of Fig. 4 ▸, and the Greek letters correspond to the slices shown in Fig. 13 ▸

A pin-hole projective model of the IR camera was calibrated and used to reproject the measured thermal fields onto a 3D mesh of the sample (Turpin *et al.*, 2021 ▸). Fig. 5 ▸ shows the thermal field at the initial state. A thermal gradient was applied along the *X*-direction, from the hot outer face of the long arm (520°C) to the ‘cold’ short arm (320°C). In operating conditions, the parts faced higher temperatures. However, the thermal gradient corresponded to magnitudes expected in the considered aeronautical parts near the clamping area. It will generate a differential dilation and the corresponding stress state. Such tests are essential to validate a modelling approach at several chosen conditions. Thus validated, the model can be used to assess sustainable loads over the entire range of service conditions.

As the set of IR images is highly redundant and the thermal field is described with a low-order polynomial field, the uncertainty on the measured temperature is as low as 0.01%. Nevertheless, model errors remain: *e.g.* the thermal model used to convert the heat flux into temperature assumes the emissivity is spatially invariant (these points are discussed in detail in the paper mentioned above). Even if the thermal field is expected to be stationary, it is measured at each tomographic acquisition step. Its variation from one step to the next, if observed, is taken into account. As the sample’s deformation is limited and rigid-body motion is low — as it is clamped to the rotating plate, which turns back to exactly the same position at the end of each scan — the IR-camera calibration can be performed once for all steps.

## 3. Processing of tomographic data

Several artefacts pollute the raw tomographic data. They have to be corrected for optimum exploitation.

**Figure 6:**
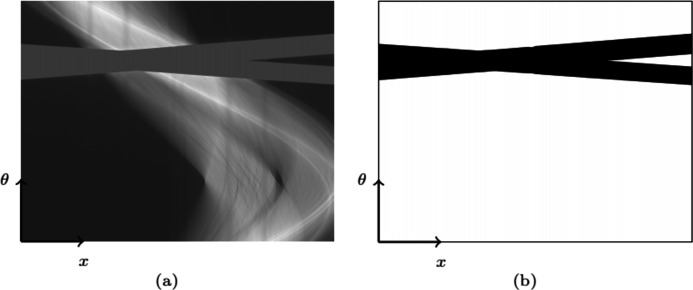
Missing-data area on the sinogram due to the columns of the loading gantry. (*a*) Sinogram slice. (*b*) Missing-data area (in black). In sinogram (*a*), the mean grey level of the available area is allocated to the missing-data area.

The steel columns of the loading gantry absorb the X-ray beam almost completely. A part of the information is thus missing in some projections, as shown in Fig. 6 ▸. Here the missing-data area represents 13% of the projections. It induces artefacts during reconstruction (*i.e.* the computation of the 3D image from the acquired radiographs) and interferes with pre-reconstruction corrections. The whole image processing workflow was implemented to master the transformations from the raw sinogram to the reconstructed volume.

The synchrotron beam is very spatially coherent. The dephasing generated passing through a material interface creates phase contrast. Phase contrast is characterized by very sharp and exaggerated grey-level variations at interfaces. This effect can be beneficial in increasing the visibility of weakly contrasting structures. However, the fringes may interfere with the quantitative interpretation of the reconstructed images. A Paganin filter reduces the fringe intensity and increases image contrast (Paganin *et al.*, 2002 ▸). The Paganin filter of length 30 µm is applied to the projections. The missing data are replaced by the mean grey level of the rest of the data [Fig. 6 ▸(*a*)].

In parallel-beam CT, the reconstruction field of view can be widened by moving the rotation axis away from the beam centre and performing the acquisition over 360° (Stock, 2008 ▸). The first half turn provides the projection of one half of the sample; the second half turn gives the rest. Then, those half projections are stitched to conform to the standard (axis-centred) tomographic geometry. Their relative positions are determined pixel-wise from the known acquisition geometry. Indeed, a more accurate positioning would have imposed interpolating one side of the projection, which is computationally expensive and useless because the Nyquist resolution is two times the pixel size. The continuity of the microstructure in the reconstruction proves that this precision is sufficient. As the beam intensity can vary a little during the acquisition, an overlap area (around the axis position) is used to balance the grey level between the two sides of the stitched projections.

**Figure 7:**
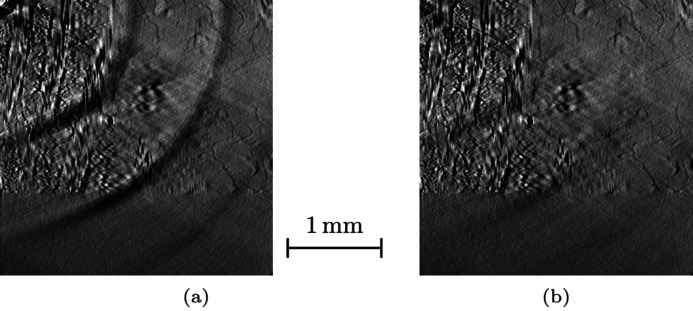
Correction of ring artefacts. Zoom on a horizontal slice of the reconstruction: the centre of rotation is at the upper left corner. The spots on the projections create dark rings in the gross reconstruction (*a*), which almost vanish on the corrected one (*b*).

The imperfections and soiling on the optical chain of the tomograph (scintillator, lens, mirror, detector, *etc*.) form spots on the projections. Those spots create ring artefacts on the reconstruction. They are always present at the same position on the projections, and thus can be revealed and corrected by summing the sinogram along the θ-axis. On this profile, the positions of defects appear as peaks. Sinogram grey levels are re-adjusted using an *x*-dependent correction, smoothing this profile. Fig. 7 ▸ shows a reconstruction before and after correction.

The most commonly used and effective reconstruction algorithm is filtered back-projection (Kak & Slaney, 2001 ▸). Nevertheless, this algorithm needs equiangular full projections. Iterative reconstruction reprojection (IRR) addresses missing-data problems (Nassi *et al.*, 1982 ▸). First, this procedure reconstructs the raw sinogram affecting an arbitrary value to the missing-data area. The reconstruction is then filtered and reprojected. The measured sinogram is completed in the missing-data area using the reprojection of the previously reconstructed volume. This loop is repeated as many times as needed. The efficiency of IRR depends a lot upon the quality of the filtering applied to the reconstructed volume. Recent work proposes a phase-field regularization based on the assumption that the sample is composed of a finite number of phases (Turpin *et al.*, 2020 ▸). Using this regularization, the number of IRR iterations to reach convergence is as low as three. The filter parameters are [with the notation of Turpin *et al.* (2020 ▸)]: α = 5, μ = 0.05μ_max_ and ξ = 1 voxel.

Three stitched scans enlarge the field of view vertically (along the *Z*-axis). The position of each scan is known pixel-wise from experimental set-up data. When stitching images, grey level continuity is assured by scaling one image such that the mean values in the overlapping region are the same.

**Figure 8:**
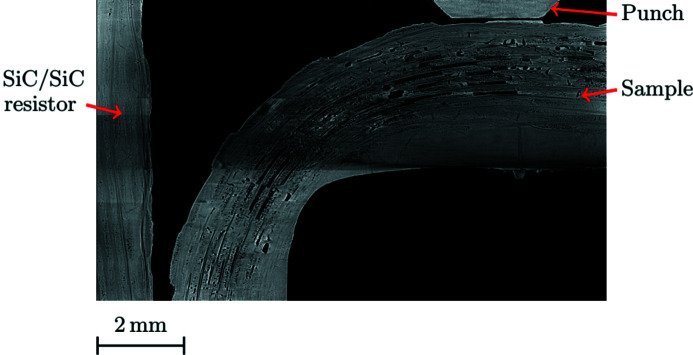
Tomograph of the sample where the beam hardening is noticeable: the part where the length crossed by the X-ray is higher appears darker.

A pink beam is used rather than a monochromatic one to increase acquisition speed. Fig. 8 ▸ displays the result of regularized-IRR. Some beam hardening occurs and is perceptible where the cross-section varies abruptly. As the SiC/SiC resistor is expected to have a relatively homogeneous grey level, it corrects this last artefact. The mean grey level of each horizontal slice of a region within the resistor is computed. The curve obtained, mean against *Z*, is smoothed with a moving average that provides the corrective factor to apply slice by slice to restore homogeneity. In practice, the middle scan is corrected before the vertical stitching.

**Figure 9:**
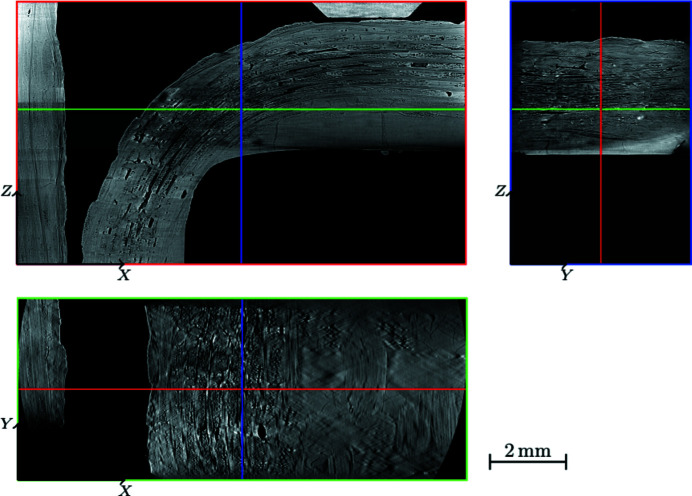
Tomographic reconstruction of the initial step after all artefact corrections.

Fig. 9 ▸ shows the processed tomographic reconstruction of the sample in its initial state. The entire zone of interest is visible: the short arm and the elbow.

The signal-to-noise ratio (SNR) can be defined from the image as the ratio between the mean grey level value inside the sample and the grey level standard deviation in an area outside of the sample. The SNR of the initial step volume is 12.3.

**Figure 10:**
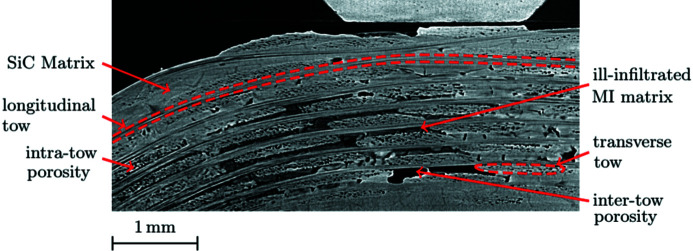
Sample microstructure: zoom on an *XZ* slice of the tomography in the initial state.

During pre-processing, some steps have to be done carefully (*e.g.* stitching) in order not to create additional artefacts. We attempted to avoid any over-correction on the zone of interest (*i.e.* inside the material). The ring artefacts do not vanish. For the phase-contrast, a global correction, such as that of Paganin’s, tends to degrade the sharpness of the images. Thus the correction is applied conservatively to avoid over-smoothing. Moreover, the phase-contrast can reveal some under-resolved details, such as intra-tow pores (Fig. 10 ▸). After corrections, the signature of artefacts present in the initial projections has been considerably reduced. They will no longer significantly influence subsequent exploitation (*e.g.* threshold determination, DVC).

## 4. Experimental results and discussion

### 4.1. Micro- and meso-structural integrity of the samples at the initial step

CT can be used to characterize the material integrity of the samples. Fig. 10 ▸ points out salient features of the tested CMC, highlighting its complex woven structure and multi-phase matrix. In the current acquisition conditions, tomography describes the mesostructure well, namely the arrangement of the tows, and allows one to distinguish some microstructural elements (*e.g.* tow section geometry or tow micro-porosity), even though their precise geometry cannot be determined. Besides tows and SiC matrix, defects are visible. Although the skin of the sample is well infiltrated (the matrix is dense and scarcely discriminated from the tows), infiltration is not perfect in bulk. Matrix pores and poorly infiltrated MI-matrix areas — which are zones filled with SiC powder — are numerous. The higher weave density results in an increased concentration of infiltration deficiencies in the elbow area. Those defects are expected to decrease the sample stiffness.

The resolution of the tomography is not sufficient to measure intra-tow porosity (fibre diameter is 12 µm and pixel size is 3.14 µm). Pore shapes are not measured accurately, but they are observed on most of the tows in Fig. 10 ▸.

The porosity of the sample can be estimated from the tomography (Maire & Withers, 2014 ▸; Jaques *et al.*, 2021 ▸; Lifton & Liu, 2021 ▸). Most porosity estimation procedures are based on more or less complicated segmentation algorithms, some providing sub-resolution precision (Smal *et al.*, 2018 ▸). In SiC/SiC materials, micro-porosity is mainly located within the tows (so-called intra-tow porosity). It is visible on the volumes (Fig. 10 ▸); the pores have the shape of a pin with a diameter that does not exceed one or a few voxels. This intra-tow porosity cannot be measured properly. However, its impact on the mechanical behaviour of the tows has already been studied numerically (Mazars, 2018 ▸). Here, we focus on the role of the mesostructure on the macroscopic damage behaviour. It is thus relevant to consider mainly mesopores which are numerous in the matrix and constitute potential crack initiation or propagation sites. A gross estimate of the porosity can be obtained by cropping and thresholding the reconstructed volumes.

**Figure 11:**
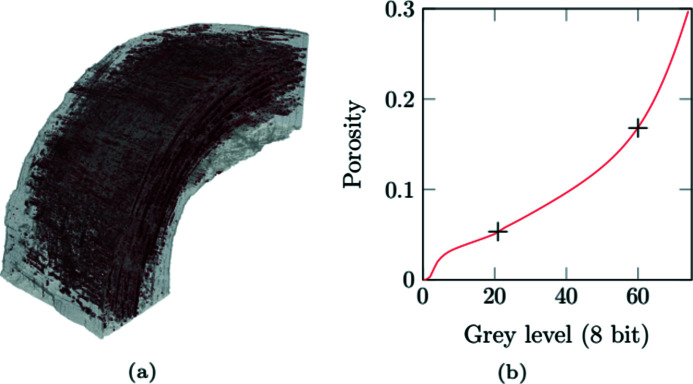
Porosity of the sample used for the high-temperature test. (*a*) Pore map (grey level threshold, 21). (*b*) Effect of the grey level threshold on the determined porosity. The two cross marks correspond to the chosen values for porosity taking into account SiC powder zones or not.

Considering the quality of the images, a unique threshold for the whole sample is suitable. The contrast between air and SiC grey levels is far higher than the noise level and — after the corrections detailed in Section 3 — higher than the effect of beam hardening. It also provides a pore map like the one depicted in Fig. 11 ▸. Such a map can be further exploited to consider the matrix defects into a mesoscale model of the sample. Nonetheless, the choice of the threshold significantly influences the evaluation of porosity. In the present case, two values are of interest. The first one focuses on actual pores and provides a lower bound on the porosity as only pores larger than a voxel are discernible. The second one, in contrast, is based on the grey-level of ill-infiltrated regions and can be used as an upper bound. The latter is mechanically relevant as the unconsolidated agglomerates of SiC powder are not expected to play a significant part. Moreover some open pores of the sample were ill-infiltrated matrix zones before machining. The curve [Fig. 11 ▸(*b*)] is simply a normalized cumulative histogram of grey levels in the sample volume. The two cross marks correspond to manually determined porosities. The curve provides an estimate of the uncertainty which can be defined as the porosity variation induced by a threshold variation of one grey level. Porosity values are 5.3% ± 0.3% without considering SiC powder zones and 17.5% ± 0.6% taking them into account for the sample used for the high-temperature test.

### 4.2. Overall behaviour

The incremental sensor of the punch actuator and the load cell provides what is hereafter called the *macroscopic response* of the sample. To take into account the compliance of the loading cell, the incremental-sensor displacement is re-adjusted using tomographic data, as explained in Appendix *A* [App appa].

**Figure 12:**
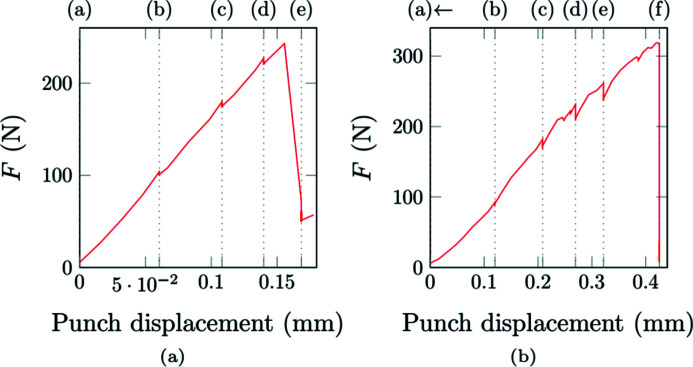
Force displacement curves of the *in situ* experiments. Letters indicate tomographic and thermographic acquisitions. The punch displacement is re-adjusted as detailed in Appendix *A* [App appa]. The test (*a*), at room temperature (20°C), was performed without heating the sample. During the test (*b*), under thermal gradient, the sample was heated, as shown in Fig. 5 ▸.

During the test at room temperature [Fig. 12 ▸(*a*)], four loading steps are performed in addition to the initial acquisition (*a*). A slight relaxation is observed during each acquisition step. The response is nearly linear up to step (*d*). Suddenly, a macroscopic crack propagates between step (*d*) and step (*e*). After step (*e*), the force still increases with the displacement, showing that the sample exhibits a residual mechanical resistance because the crack does not propagate throughout the entire thickness. A post-mortem observation of the sample reveals that the main crack initiated at the outer surface of the corner elbow and propagated radially.

During the test under thermal gradient, the resistor is maintained at about 1100°C while the cold point of the sample remains at about 300°C. Five acquisition steps are performed. The sample rupture occurs abruptly during step (*f*). Relaxation phenomena are initially of small amplitudes, comparable with the test performed at room temperature. They increase after step (*d*) when a loss of rigidity is observed. The apparent rigidity is lower than the one observed at room temperature. Such a difference is unexpected. Indeed, SiC/SiC mechanical properties do not vary much with temperature. The two samples were machined from the same part and were selected because they exhibited an equivalent mesostructure (same type and equivalent distribution of defects).

Tomograms do not reveal much change in the material morphology. The considered temperatures are not high enough to induce oxidation or substantial softening of some phases. Such phenomena are not the focus of this study. The objective is to acquire data that will be used to perform DVC and then calibrate a model, taking into account the local stress multi-axiality.

### 4.3. Observation of damage

**Figure 13:**
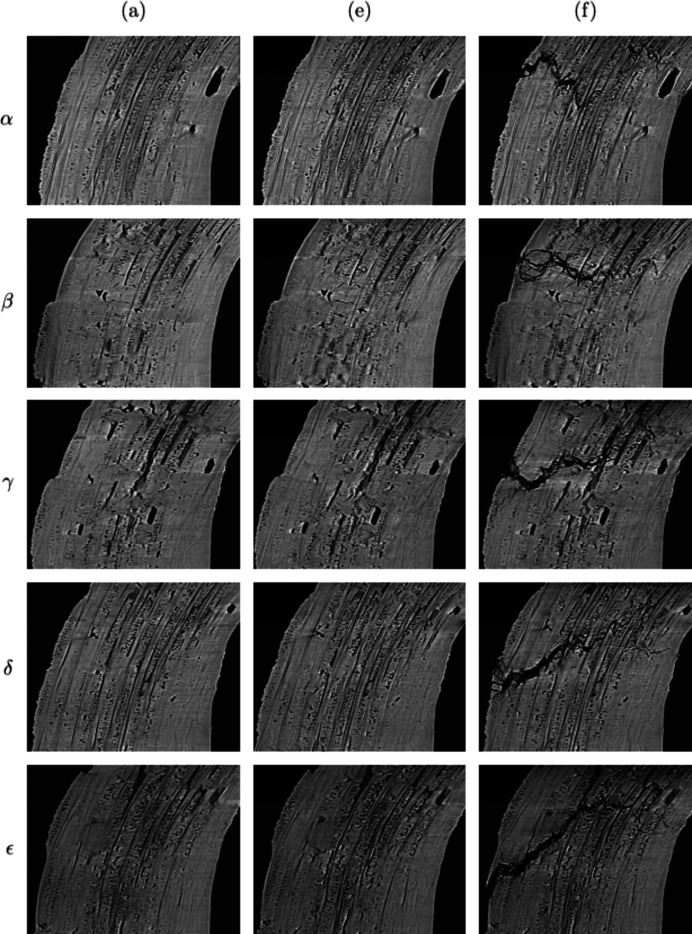
Crack path for the high-temperature test for slices taken every 630 µm (the Greek letters correspond to the slice positions as plotted in Fig. 5 ▸). (*a*) Initial state. (*e*) At last step before failure. (*f*) After failure.

The combination of the sample geometry, its structure and the loading mode tends to concentrate the stress in a given area. Contrary to regular loading (*e.g.* tensile test), cracking is localized and occurs rather abruptly. No network of micro-cracks is observed in the volume. The main crack initiates in the outer skin of the elbow, and it is located close to its junction with the clamped arm (Fig. 13 ▸). It coincides with the area where a macroscale thermo-elastic finite-element simulation (*i.e.* considering a homogeneous, orthotropic material) predicts that the major principal stress is maximal. The part geometry thus plays a first-order role in damage localization. Then the crack propagates within the bulk of the sample, being deviated by the woven structure as shown from its irregular path.

The crack propagates mainly within tows or at the interface between tows and matrix. From Fig. 13 ▸, one may infer that the main crack initiates from the sample outer skin, breaking through transverse and longitudinal tows. Locally, the crack path follows the microstructure. Starting from a free surface, the crack tends to go through the most prominent matrix pores and the smaller porosities surrounding the tows. It is also influenced by the ill-infiltrated matrix areas, either following pre-existing shrinkage cracks or the borders of those areas. This observation is consistent with this local path minimizing the surface energy required to propagate the crack front between pores. The complex distribution of defects influences the large-scale geometry of the crack surface. The coalescence of guided cracks induces a relatively high ratio between crack surface and volume compared with common cracks in homogeneous brittle materials. In bulk transverse tows, the crack exhibits multiple branching. Crossing longitudinal tows, it is deviated and leads to fibre debonding within the tows or at the matrix/tow interface. Variability in the matrix/tow interface behaviour exists. On the one hand, some interfaces are weak — as they were designed to be — some tows around the crack path show a highly damaged interface with the matrix, and some fibres appear disconnected from each other (as in the right-hand side of slices δ or ε). On the other hand, some interfaces remain strong, and the crack cuts straight across tows (as in slice β). All observed damaged longitudinal tows broke at a single position.

At step (*f*), the crack opening is large, up to 135 µm, even if the crack does not propagate through the entire thickness of the sample. The damaged area thickness is far more extended than in a brittle material. Outside of the damaged area, no evidence of damage is visible in the reconstructed volume. Within the spatial resolution, failure appears to be sudden and, hence, the overall behaviour brittle. This is in line with the design of such parts where ultimate loads coincide with the ‘first damage’ that can be detected.

### 4.4. Exploitability of the data by DVC

Digital Volume Correlation (DVC) is a numerical method to compute the displacement field between two images based on grey level conservation assumption. A physical point is assumed to keep the same grey level throughout the test. In the present case of synchrotron tomography, deviations from the grey level conservation assumption are only induced by artefacts and noise in the images.

Most of the artefacts are indeed stationary in the reference frame of the image, *e.g.* ring artefacts, discontinuities at the stitched surface, spatial inhomogeneity of the beam and, at the first order, the artefacts induced by the columns of the loading device. In the studied material, which is very stiff, the magnitude of displacement is low, and beam hardening is not expected to vary much from one image to the next. A proper correction of all these artefacts, at least inside the sample, is required so as not to interfere with DVC analysis.

The uncertainty on the displacement field measured by DVC is proportional to the noise level of the images. This noise level remains high for tomographic imaging even after carefully choosing the acquisition conditions to keep it low. Typically, extending the exposure time decreases the noise. Conversely, the presence of any element reducing the dynamics of the image will increase it. In our experiment, there are no X-ray windows around the sample, and the noise level of the projections is low for an *in situ* experiment. The choice of artefact filters which do not downgrade the image quality is also essential.

The support (source of contrast) of the correlation is the texture of the image. Here the texture is provided by the textile fabric and the heterogeneity of the matrix. The contrast between those textural elements must be significantly higher than the noise level to measure a relevant field. In the studied material, this is particularly challenging. The contrast between the matrix and the fibre, SiC and SiC, is necessarily weak. The acquisition conditions have to be tuned finely. This need for texture contrast between fabric and matrix is also the main reason to work with such high-resolution images. In well infiltrated areas of the material, where the matrix is homogeneous, the only distinguishable texture comes from the intra-tow porosity.

These three observations allow us to confirm that these images are exploitable by DVC, which has been confirmed by preliminary tests.

## 5. Conclusion

An experiment was carried out to characterize a sample with a non-conventional geometry submitted to complex loading. The SiC/SiC samples presented here have an L-shape. The geometry induces stress concentration and drives the failure location at the first order. The failure is brittle, as is the propagation of the main crack. There is no evidence of a network of diffuse micro-cracks. The *in situ* tomographic study gives keys to understanding the physical phenomena leading to the mechanical behaviour of the sample.

This kind of *complex* test has two significant advantages: it is close to operating conditions and provides original loading configurations. Its inhomogeneity, however, requires a full-field analysis to capture a large zone of interest where the behaviour (here, cracking and damage) has to be characterized. As the coupling between the thermal (gradient) loading, the contact forces applied onto the sample, and the sample geometry induce a wide variety of local loading configurations, this test has the potential to discriminate between different models or different parameters in a given model. The long-term target is to automatically identify the constitutive law based on these full-field measurements. Utmost care preserves the data quality guiding the design of the high-temperature and mechanical testing device and the image processing procedure to minimize the detrimental effects of numerous artefacts.

Experimental constraints lead to difficult tomographic acquisition conditions. Careful image processing for tomographic reconstruction, namely regularized-IRR, circumvents missing data artefacts. Image processing ultimately provides high-quality reconstructed volumes. The texture provided by the woven fabric and the heterogeneity of the matrix will be a good support for DVC. The corrected volumes show only weak artefacts, which should not interfere much with full-field measurements. The relatively low noise level of the images should lead to an acceptable uncertainty. However, due to the heterogeneous texture, the spatial resolution of the displacement field measurement with DVC alone is expected to be limited. For that reason, a thermo-mechanical integrated-DVC algorithm is being developed to identify the material properties of the mesoscale model of the sample and will be discussed in a companion paper (Turpin *et al.*, 2022 ▸). A direct identification procedure is an elegant way to bypass the spatial resolution problem.

The experimental device was designed to be versatile enough to use other sample geometries. It has been used to test T-shaped samples or corners under four-point bending. The processing protocol remains the same. The tests could also be extended to studying other composite materials, which usually should be much less challenging than CMCs. Future thermo-mechanical tests may be designed: (i) either to validate modelling so that geometry or thermo-mechanical loading are optimized to be as sensitive as possible to chosen parameters, which are difficult to access; or (ii) to investigate ultimate loads for experimental validation of the admissible service (or extreme) conditions.

These two approaches aim to understand the material behaviour and check the reliability of such complex parts for multiaxial thermo-mechanical loading. Coupling inhomogeneous thermal and mechanical full-field, this study widens the scope of X-ray CT *in situ* tests to more complex configurations.

## Figures and Tables

**Figure 14:**
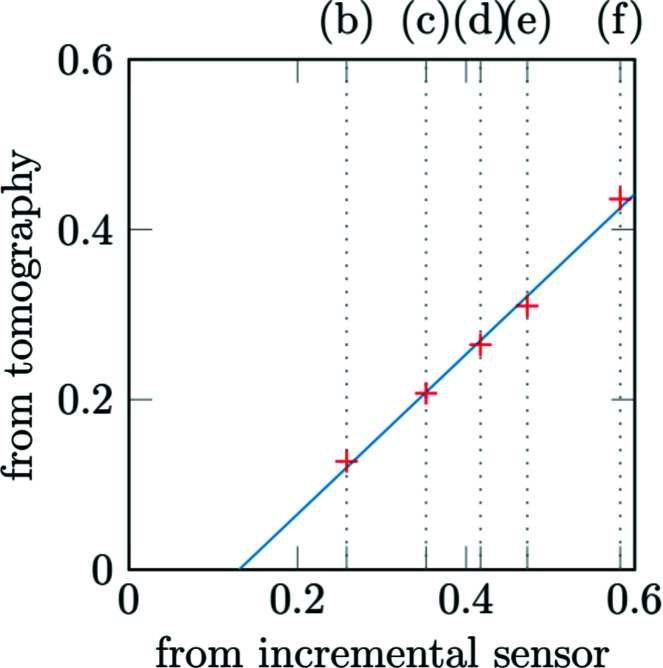
Re-adjustment of the punch displacement (mm).

## Appendix A Adjustement of the measured punch displacement using tomographic images

The displacement of the punch is not directly measured during the experiment. Only the incremental sensor of the motor is recorded continuously. More accurate data are available at each acquisition step: the position of the punch is known from tomography. Its position can be plotted against the estimated one (Fig. 14 ▸). Assuming that the error on the sensor displacement is mainly due to the compliance of the loading machine, the transformation from the sensor to tomographic displacement can be adjusted to an affine law. It is determined from a linear regression on the few available points. This transformation is used to correct the entire displacement curve.

The displacement data plotted in Fig. 12 ▸ were corrected using the above calibration.

Moreover, the displacement of the visible part of the long arm of the sample can also be computed on a small region of interest using a simplified DVC, allowing only for rigid-body motion. It should be used to assess the absence of sample slips in the grips. In the present case, no evidence of significant slip was found. The zone of interest moves as it is located 1 cm above the clamped zone. The vertical displacement in this area is one order of magnitude lower than the punch displacement. It is nearly proportional to it [considering acquisition steps (*b*) to (*e*) for the high-temperature experiment, Fig. 12 ▸(*b*)].
